# Risk Model for Laryngeal Complications Prediction in Chinese Patients After Coronary Artery Bypass Grafting

**DOI:** 10.21470/1678-9741-2023-0424

**Published:** 2024-09-06

**Authors:** Jiangyun Peng, Yinghong Zhang, Xuan Liu, Xue Feng, Zijun Yin, Yanhong Hu, Wen Zhang, Jing Zhang, Jingping Li

**Affiliations:** 1 Institute of Nursing Research, Hubei Province Key Laboratory of Occupational Hazard Identification and Control, School of Medicine, Wuhan University of Science and Technology, Wuhan, Hubei, People’s Republic of China; 2 Department of Cardiology, Wuhan Asia Heart Hospital affiliated to Wuhan University of Science and Technology, Wuhan, Hubei, People’s Republic of China; 3 Department of Otolaryngology, Wuhan Puren Hospital, Wuhan, Hubei, People’s Republic of China

**Keywords:** Coronary Artery Bypass, Laryngeal Complications, Risk Factors, Deglutition Disorders, Larynx

## Abstract

**Introduction:**

The aim of this study was to identify perioperative risk factors of laryngeal symptoms and to develop an implementable risk prediction model for Chinese hospitalized patients undergoing coronary artery bypass grafting (CABG).

**Methods:**

A total of 1476 Chinese CABG patients admitted to Wuhan Asian Heart Hospital from January 2020 to June 2022 were included and then divided into a modeling cohort and a verification cohort. Univariate analysis was used to identify laryngeal symptoms risk factors, and multivariate logistic regression was applied to construct a prediction model for laryngeal symptoms after CABG. Discrimination and calibration of this model were validated based on the area under the receiver operating characteristic (ROC) curve and the Hosmer-Lemeshow (H-L) test, respectively.

**Results:**

The incidence of laryngeal symptoms in patients who underwent CABG was 6.48%. Four independent risk factors were included in the model, and the established aryngeal complications risk calculation formula was Logit (*P*) = −4.525 + 0.824 × female + 2.09 × body mass index < 18.5 Kg/m^2^ + 0.793 × transesophageal echocardiogram + 1.218 × intensive care unit intubation time. For laryngeal symptoms, the area under the ROC curve was 0.769 in the derivation cohort (95% confidence interval [CI]: 0.698-0.840) and 0.811 in the validation cohort (95% CI: 0.742-0.879). According to the H-L test, the *P*-values in the modeling group and the verification group were 0.659 and 0.838, respectively.

**Conclusion:**

The prediction model developed in this study can be used to identify high-risk patients for laryngealsymptoms undergoing CABG, and help clinicians implement the follow-up treatment.

## INTRODUCTION

There is an increasing incidence of coronary artery disease (CAD) nationwide, and therapies for CAD have evolved, including surgeries, interventions, and medications^[^1^]^. Coronary artery bypass grafting (CABG) surgery is often considered a high-risk procedure, associated with a 30-day morbidity and mortality rate up to 14.0% and 2.0%, respectively^[^2^]^. However, postoperative complications of CABG including respiratory failure, stroke, urinary tract infections, and renal failure remain common^[^2^]^.There is growing concern on the complications of laryngeal injury, including swallowing disorders (dysphagia) and voice disorders (dysphonia)^[^3^]^.

Laryngeal complications (LCs) include dysphagia/aphasia, dysphonia/aphonia, and vocal cord paralysis^[^3^]^, which may occur after endotracheal intubation, esophagectomy, anterior cervical spine surgery, thyroidectomy, and cardiac surgery. Several studies have shown that the occurrence of LCs is 3%-62% after extubation^[^4^]^, 1.96% after esophagectomy^[^5^]^, 32.4% after anterior cervical spine surgery^[^6^]^, as well as the incidence in cardiac surgery ranges from 1.5 to 1.8%^[^3^]^. Specifically, the rate of LCs almost increases every year in cardiac surgery (2010-2017)^[^3^]^.

Once LCs occurs, it increases the secondary complications such as pneumonia and aspiration, and then brings malnutrition, longer length of hospital stay, and higher total hospital costs^[^5^, ^7^, ^8^]^. Postoperative complications increase among patients with LC^[^9^]^. In addition, because the early clinical symptoms of patients with LCs are not easy to be found by clinicians^[^9^]^ and LCs were characterized by transient dysphonia and dysphagia^[^10^]^, little attention has been paid to the laryngeal consequences after CABG^[^10^]^. However, assessments of swallowing and voice function are not conducted as a routine examination^[^9^]^. It appears that clinicians should increase the awareness of post-extubation dysphagia and implement bedside screening^[^11^]^. In addition, early examination and treatment could reduce patients’ costs^[^8^]^.

In recent years, major studies have focused on the risk factors of LCs and postoperative treatment among patients after neck surgery and extubation, and there are far less investigations in patients who underwent CABG. A good prediction model can help identify the risk of LCs^[^12^]^, therefore, the main objective of our study was to develop a risk prediction model of LCs for Chinese patients undergoing CABG to help clinical staff screen patients with LCs and implement effective interventions.

## METHODS

### Sample

Our study was approved by Wuhan University of Science and Technology (22Z107). Data were collected from patients who underwent CABG from January 2020 to June 2022 in Wuhan Asian Heart Hospital affiliated to Wuhan University of Science and Technology. Inclusion criteria were patients aged ≥ 18 years and patients who underwent CABG. Exclusion criteria were patients with incomplete data and patients with previous history of laryngeal problems. Among 1476 cases, 844 patients from January 2020 to June 2021 were used as the derivation cohort, while 632 patients from July 2021 to June 2022 were used as the validation cohort. Our study was approved by the Medical Ethics Committee of Wuhan University of Science and Technology (reference number: 202217)

### Methods

Data were collected via retrospective review of the electronic record system. Dysphagia, dysphonia/aphonia, and vocal cord paralysis were identified by doctors from patients’ clinical manifestations and examinations, such as laryngoscopic evaluation. For patients without symptoms, dysphagia/aphasia was screened by the swallowing test first^[^10^]^, and grades 3-5 were defined as dysphagia. Then patients were further diagnosed according to clinical examinations such as laryngoscopic evaluation. The classification criteria of swallowing test are as follows:

Grade 1: capable of drinking (swallow) all water in one going with no side effect.
Grade 2: capable of drinking all water by two swallows without causing coughing.
Grade 3: capable of swallowing in one go but accompanied by coughing.
Grade 4: need multiple swallows, and also have coughing.
Grade 5: need multiple swallows accompanied with frequent coughing.

### Variable

A total of 23 variables were included in this study — (1) general variable: age, sex, body mass index (BMI), smoking and drinking history, admission, and insurance type; (2) perioperative variable: transesophageal echocardiogram (TEE), combined surgery type, diabetes, heart valve disease, re-entry to intensive care unit (ICU), number of coronary artery bypass grafts, ICU tracheal intubation time, tracheal tube size, tracheal intubation depth, endotracheal cuff pressure (CP), operative time, surgery method, cerebrovascular lesion, chronic lung disease, preoperative myocardial infarction, history of percutaneous coronary intervention, and length of stay in ICU.

### Statistics Analysis

Data were analyzed using IBM Corp. Released 2019, IBM SPSS Statistics for Windows, version 26.0, Armonk, NY: IBM Corp., and we considered *P*<0.05 to be statistically significant. Patients’ baseline characteristics are described as frequencies and percentages. Comparisons among groups were performed using chi-square tests. We used multiple logistic regression to identify risk predictors and construct the prediction model. The discrimination of the risk prediction model was assessed by the area under the receiver operating characteristic curve, and Hosmer-Lemeshow (H-L) goodness of fit test was used to validate the calibration.

## RESULTS

### Basic Characteristics

A total of 1476 patients who underwent CABG were included for analysis, 68.43% of whom were male. Patients were aged from 38-83 years, with an average age of 63 years. The average number of grafts was 3 + 1.13 (mean + standard deviation). Baseline covariates are shown in Table 1.

**Table 1:** Baseline characteristics in derivation cohort.

Variable	No diagnosed LCs	LCs	P-value
	(N=798)	(N=46)	
Age (years)			0.073
< 60	257 (32.2)	9 (19.6)	
≥ 60	541 (67.8)	37 (80.4)	
Sex			0.002
Male	554 (69.4)	22 (47.8)	
Female	244 (30.6)	24 (52.2)	
Body mass index (Kg/m^2^)			0.008
< 18.5	24 (3.0)	6 (13.0)	
18.5-24.9	416 (52.1)	20 (43.5)	
≥ 24	358 (44.9)	20 (43.5)	
Admission type			0.576
Outpatient	523 (65.5)	32 (69.6)	
Emergency	275 (34.5)	14 (30.4)	
Smoking history			0.028
Yes	393 (49.2)	15 (32.6)	
No	405 (50.8)	31 (67.4)	
Alcohol history			0.646
Yes	161 (20.2)	8 (17.4)	
No	637 (79.8)	38 (82.6)	
Insurance type			0.378
Private insurance	145 (18.2)	6 (13.0)	
Other	653 (81.8)	40 (87.0)	
Use ofTEE			0.001
Yes	371 (46.5)	33 (71.7)	
No	427 (53.5)	13 (28.3)	
Valve disease			< 0.001
Yes	93 (11.7)	15 (32.6)	
No	705 (88.3)	31 (67.4)	
Diabetes			0.028
Yes	243 (30.5)	7 (15.2)	
No	555 (69.5)	39 (84.8)	
A history of PCI			0.511
Yes	80 (10.0)	6 (13.0)	
No	718 (90.0)	40 (87.0)	
Surgery type			< 0.001
Isolated CABG	573 (71.8)	18 (39.1)	
Non-isolated CABG	225 (28.2)	28 (60.9)	
Re-entry to ICU			0.016*
Yes	2 (0.3)	2 (4.3)	
No	796 (99.7)	44 (95.7)	
Prior myocardial infarction			0.138
Yes	154 (19.3)	13 (28.3)	
No	644 (80.7)	33 (71.7)	
Operation time (hours)			0.001
< 5.03	452 (56.6)	15 (32.6)	
≥ 5.03	346 (43.4)	31 (67.4)	
ICU endotracheal intubation time (hours)			< .001
< 14.96	540 (67.7)	18 (39.1)	
≥ 14.96	258 (32.3)	28 (60.9)	
Surgery method			0.392*
Sternotomy	773 (96.9)	46 (100.0)	
Minimally invasive	25 (3.1)	0 (0.0)	
Cerebrovascular lesion			0.758
Yes	99 (12.4)	5 (10.9)	
No	699 (87.6)	41 (89.1)	
Chronic lung diseases			0.429*
Yes	69 (8.6)	2 (4.3)	
No	729 (91.4)	44 (95.7)	
Endotracheal tube size (mm)			0.019
< 8.0	546 (68.4)	39 (84.8)	
≥ 8.0	252 (31.6)	7 (15.2)	
Number of CABG (root)			0.284
< 3	213 (26.6)	9 (19.6)	
≥ 3	584 (73.2)	37 (80.4)	
Endotracheal tube depth (cm)			0.121
< 23	334 (41.9)	14 (30.4)	
≥ 23	461 (57.8)	32 (69.6)	
Endotracheal cuff pressure (cmH_2_O)			0.437
< 26	153 (19.2)	11 (23.9)	
≥ 26	642 (80.5)	35 (76.1)	

CABG=coronary artery bypass grafting; ICU=intensive care unit; LCs=laryngeal complications; PCI=percutaneous coronary intervention; TEE=transesophageal echocardiogram.

* Fisher’s exact test

### Laryngeal Complications’ Outcomes

LCs were present in 95 (6.48%) patients after CABG, including dysphagia (3.32%, n=49), dysphonia (2.37%, n=35), and dysphagia and dysphonia (0.75%, n=11). Dysphagia is most common among laryngeal injuries in CABG patients. There were more than half of the LC patients over 65 years (59%).

Chi-square tests showed that there were significant differences with respect to sex, BMI, smoking, TEE, valve disease, diabetes, combined surgery type, re-entry to ICU, operation time, ICU tracheal intubation time, and tracheal tube size between LC and non-LC patients (*P*<0.05) (Table 1). Multivariate analysis revealed that sex, BMI, TEE, tracheal tube size, and ICU tracheal intubation time differed significantly between the LC and non-LC groups (Table 2).

**Table 2:** Multivariate analysis of predictors of LCs after CABG in derivation cohort.

**Variable**	**B**	**Wald**	***P*-value**	**OR**	**95% CI**	
					**Lower limit**	**Upper limit**
Sex (female)	0.824	5.56	0.018	2.279	1.149	4.519
BMI (Kg/m^2^)		16.731	< 0.001			
< 18.5	2.09	16.636	< 0.001	8.083	2.961	22.065
≥ 24	0.374	1.187	0.276	1.453	0.742	2.848
Use ofTEE	0.793	4.931	0.026	2.209	1.097	4.447
Tracheal tube size ≥ 8.0 (mm)	−0.568	1.472	0.225	0.566	0.226	1.419
ICU endotracheal intubation time ≥ 14.96 (hours)	1.218	12.139	< 0.001	3.380	1.704	6.706
Constant	−4.525	99.818	< 0.001	0.011		

BMI=body mass index; CABG=coronary artery bypass grafting; CI=confidence interval; ICU=intensive care unit; LCs=laryngeal complications; OR=odds ratio; TEE=transesophageal echocardiogram

### Multivariate Model

Table 2 displays results of the multivariate regression model. The established LCs risk calculation formula was Logit (*P*) = −4.525 + 0.824 × female + 2.09 × BMI < 18.5 Kg/m^2^ + 0.793 × TEE + 1.218 × ICU intubation time.

### Model Performance

**Fig. 1:**
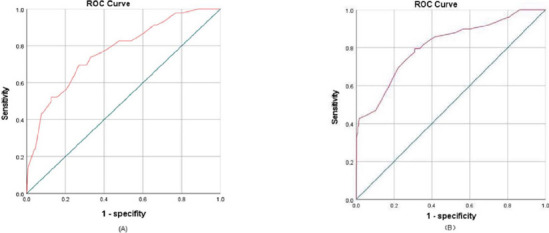
Area under the receiver operating characteristic (ROC) curve plots for prediction model fitted on: A) derivation samples; B) validation samples.

**Fig. 2:**
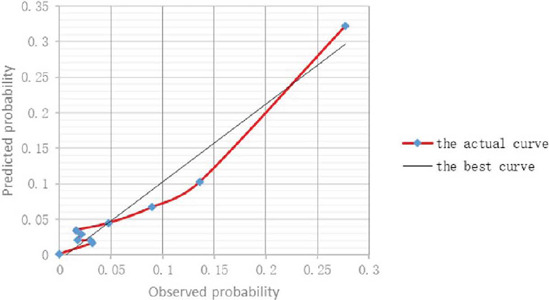
Calibration curve of predicted probability and actual probability of laryngeal complications after coronary artery bypass grafting.

We evaluated the performance of the derived model on 632 CABG cases. The C-statistic for the incidence of LCs in the derivation and validation cohorts was 0.769 (95% confidence interval [CI]: 0.698-0.840) and 0.811 (95% CI: 0.742-0.879), respectively (Figure 1). As showed in H-L test, the *P*-values in the modeling group and the verification group were 0.659 and 0.838, respectively (Figure 2).

## DISCUSSION

In our study, the prevalence of LCs after CABG surgery was 6.48% (n=1476), including dysphagia (3.32%, n=49), dysphonia (2.37%, n=35), and dysphagia and dysphonia (0.75%, n=11), which was higher than the incidence reported in recent studies. Verma et al.^[^3^]^ showed that the incidence of LCs was 1.7% based on the Nationwide Readmissions Database, which was consistent with the frequency of vocal fold paralysis reported following esophagectomy^[^5^]^. This study included approximately 2,319,628 patients with cardiac surgery and conducted International Classification of Diseases 9/10 diagnosis codes to identify LCs. In this study, LCs included vocal fold paralysis/paresis, dysphagia, dysphonia, aphagia, and aphonia, whereas in our study, just dysphagia, dysphonia, and dysphagia and dysphonia were included. Our study was conducted in an Asian hospital, and LCs were identified through patients’ symptoms, the screening test, and clinical examinations. These national sample studies may underestimate the incidence of LC due to a lack of standardized screening procedures^[^3^, ^5^]^. These discrepancies can be explained by methodological differences among studies^[^5^]^.

Although a meta-analysis suggested that the rate of oropharyngeal dysphagia after receiving mechanical ventilation ranges from 3% to 62%^[^4^]^, McIntyre et al.^[^4^]^ pointed out that significant variation exists in the reported percentages between different dysphagia assessments. The incidence of post-extubation dysphagia was 42% from endoscopic assessment, 43% through clinical swallowing examination, 41% based on patient self-report, and 62% with the videofluoroscopy. We evaluated the dysphagia of every post-CABG procedure patient after leaving the ICU, according to the water swallowing level. Furthermore, they reached a conclusion that the rates of dysphagia did not have a significant association with different assessment methods, variable patient populations, participant recruitment methods, variable time of dysphagia assessments, or median duration of intubation^[^4^]^. However, variation in study design and different sensitivity and specificity of assessment ways may be the reason of the wide CI in patients who performed dysphagia after extubation^[^4^]^.

The predictive model for LCs incorporated four risk factor variables: sex (female), BMI (low BMI and high BMI), use of TEE, and ICU intubation time, including preoperative and perioperative variables. This hints that both preoperative and perioperative management are great necessary to prevent the occurrence of LCs. Moreover, patients with longer intubation time were prone to have LCs. Previous study revealed a relationship between low BMI and malnutrition^[^8^]^. Feng et al.^[^7^]^ have shown that dysphagia and poor nutritional status seem to have strong interrelationships in the prognosis and mortality. This is the same as the conclusion in a Korean Nationwide Study that dysphagia raises the risk of malnutrition, poor prognostic outcomes, and mortality^[^8^]^.

Female sex is another predictor of the LCs. However, in our study, there were more males than females undergoing CABG surgery. It is consistent with the National database outcomes which reported that only 17.7% of all patients who underwent CABG were female^[^14^]^. Because the recognition of cardiovascular disease is later in women, it caused the delay of treatment for female patients^[^14^]^. Meanwhile, this study also claimed that females following cardiac surgery were older and had more comorbidities than males, which may account for their poorer outcomes.

The use of TEE is very common in cardiac surgery^[^15^]^. It is also identified as a predictor of LCs. The optimal CP suggested is between 20 and 30 cmH_2_O, but the current study demonstrated that the CP were elevated after TEE probe insertion^[^16^]^. Therefore, in patients with hemodynamic instability undergoing on-pump cardiac surgery, tracheal hypoperfusion is more likely to occur^[^16^]^. Moreover, laryngoscope-assisted probe placement can reduce trauma to the laryngeal soft tissue^[^15^]^. In addition, ICU intubation time was examined as a predictive factor for LCs, which was constant with the previous study in patients receiving prolonged endotracheal intubation after cardiac surgery. The occurrence of cerebrovascular stroke, sepsis, and prolonged endotracheal intubation were identified to be the independent predictors of dysphagia^[^17^]^. Furthermore, it has been reported that both dysphagia and longer endotracheal intubation were independent risk factors for delaying recovery to a normal oral diet, thus, delaying discharge and leading to poor nutrition consequences.

This predictive model indicates that strengthening preoperative and perioperative management and modifying controllable factors of CABG patients may prevent CABG patients from laryngeal injury. Patients with low BMI may have poor nutrition conditions^[^8^]^. The current study suggests the intervention of combining nutritional management and high-energy intake to avoid post-extubation complications in patients after cardiovascular surgery^[^18^]^.

Furthermore, nurses, dental hygienists, and speech-language pathologists could improve dysphagia outcomes by implementing multidisciplinary oral care interventions^[^18^]^. Feng et al.^[^7^]^ also pointed that stroke patients after conducting the intensive exercise-based swallow rehabilitation program may have less malnutrition and aspiration pneumonia when compared to patients without intervention.

### Limitations

The strength of this study is that it is the first to study risk factors and develop a prediction model in patients who underwent CABG. However, limitations of this study need to be acknowledged. First, because our study is a single-center retrospective study, the populations of LCs are slightly limited, and we cannot identify the severity of dysphagia. What’s more, according to the present studies, because there is lack of unified criteria to identified LCs, further research is required to develop a comprehensive best treatment, which would be an advancement in understanding for the multidisciplinary team, enabling effective collaboration to prevent the occurrence of LC and optimize quality of life for Chinese CABG patients. Therefore, the model needs further multi-center validation. Videofluoroscopy and fiberoptic endoscopy are suggested as the gold standard methods, but these are invasive and require specialized staff and facilities, and the reliability is poor in the absence of assessor training^[^13^]^. Given that patients after CABG are weak and need to stay in bed for a long time, it is difficult to complete the “gold standard” screening, and the required facilities may not be available in all units at the bedside^[^9^, ^19^]^. Therefore, dysphagia needs a standard screening and should be included in patient education and surgical consent^[^20^]^. Surgical strategies are also associated with the increasing risk of dysphagia^[^20^]^. The multifactorial nature in this high risk, complex population may explain the lack of optimal assessment and management approaches to direct clinicians. Additionally, future prospective studies could explore personalized strategies to reduce the rate of LCs. Although this study has such limitations, the prediction model can be used in Chinese patients undergoing CABG, in order to implement the assessment of diet strategy.

## CONCLUSION

In this study, we developed a predictive risk model of LCs for patients who underwent CABG, and it could be helpful for clinicians in identifying high-risk patients at the early stage and preventing possible adverse complications such as pneumonia caused by LCs.

## Abbreviations, Acronyms & Symbols

BMI: Body mass index
CABG: Coronary artery bypass grafting
CAD: Coronary artery disease
CI: Confidence interval
CP: Cuff pressure
H-L: Hosmer-Lemeshow
ICU: Intensive care unit
LC_S_: Laryngeal complications
OR: Odds ratio
PCI: Percutaneous coronary intervention
ROC: Receiver operating characteristic
TEE: Transesophageal echocardiogram

## References

[1] Duggan JP, Peters AS, Trachiotis GD, Antevil JL (2022). Epidemiology of coronary artery disease. Surg Clin North Am.

[2] Montrief T, Koyfman A, Long B (2018). Coronary artery bypass graft surgery complications: a review for
emergency clinicians. Am J Emerg Med.

[3] Verma A, Hadaya J, Tran Z, Dobaria V, Madrigal J, Xia Y (2022). Incidence and outcomes of laryngeal complications following adult
cardiac surgery: a national analysis. Dysphagia.

[4] McIntyre M, Doeltgen S, Dalton N, Koppa M, Chimunda T (2021). Post-extubation dysphagia incidence in critically ill patients: a
systematic review and meta-analysis. Aust Crit Care.

[5] Crowson MG, Tong BC, Lee HJ, Song Y, Harpole DH, Jones HN (2018). Prevalence and resource utilization for vocal fold
paralysis/paresis after esophagectomy. Laryngoscope.

[6] Paziuk T, Henry T, Koons K, Conaway W, Mangan J, Hilibrand A (2022). Dysphagia and satisfaction following anterior cervical spine
surgery: a prospective observation trial. Clin Spine Surg.

[7] Feng MC, Lin YC, Chang YH, Chen CH, Chiang HC, Huang LC (2019). The mortality and the risk of aspiration pneumonia related with
dysphagia in stroke patients. J Stroke Cerebrovasc Dis.

[8] Ko N, Lee HH, Sohn MK, Kim DY, Shin YI, Oh GJ (2021). Status of dysphagia after ischemic stroke: a Korean nationwide
study. Arch Phys Med Rehabil.

[9] Black RJ, Novakovic D, Plit M, Miles A, MacDonald P, Madill C (2021). Swallowing and laryngeal complications in lung and heart
transplantation: etiologies and diagnosis. J Heart Lung Transplant.

[10] Shinn JR, Kimura KS, Campbell BR, Sun Lowery A, Wootten CT, Garrett CG (2019). Incidence and outcomes of acute laryngeal injury after prolonged
mechanical ventilation. Crit Care Med.

[11] Zuercher P, Moret CS, Dziewas R, Schefold JC (2019). Dysphagia in the intensive care unit: epidemiology, mechanisms,
and clinical management. Crit Care.

[12] Li M, Zhang L, Xia D, Chu W, Zhao Y (2022). Construction of a risk prediction model for gastrointestinal
complications in patients after cardiac surgery and its prediction
effect. Chinese Journal of Nursing.

[13] Ramsey DJ, Smithard DG, Kalra L (2003). Early assessments of dysphagia and aspiration risk in acute
stroke patients. Stroke.

[14] Dixon LK, Dimagli A, Di Tommaso E, Sinha S, Fudulu DP, Sandhu M (2022). Females have an increased risk of short-term mortality after
cardiac surgery compared to males: insights from a national
database. J Card Surg.

[15] Patel KM, Desai RG, Trivedi K, Neuburger PJ, Krishnan S, Potestio CP (2022). Complications of transesophageal echocardiography: a review of
injuries, risk factors, and management. J Cardiothorac Vasc Anesth.

[16] Maddali MM, Al Hadifi TSM, Sathiya PM, Jose S (2022). The Effect of intraoperative transesophageal echocardiography
probe placement on the endotracheal tube cuff pressure in adult patients
undergoing on-pump cardiac surgery. J Cardiothorac Vasc Anesth.

[17] Barker J, Martino R, Reichardt B, Hickey EJ, Ralph-Edwards A (2009). Incidence and impact of dysphagia in patients receiving prolonged
endotracheal intubation after cardiac surgery. Can J Surg.

[18] Ogawa M, Satomi-Kobayashi S, Hamaguchi M, Komaki K, Izawa KP, Miyahara S (2023). Postoperative dysphagia as a predictor of functional decline and
prognosis after undergoing cardiovascular surgery. Eur J Cardiovasc Nurs.

[19] Chen Zhao, Yuanyuan Yu, Xuhui Wang (2020). Screening and clinical features of dysphagia and aspiration in
elderly patients. J Chin Rehab.

[20] Nguyen S, Sherrod BA, Paziuk TM, Rihn JA, Patel AA, Brodke DS (2022). Predictors of dysphagia after anterior cervical discectomy and
fusion: a prospective multicenter study. Spine (Phila Pa 1976).

